# Repetitive Transcranial Magnetic Stimulation of the Brain Region Activated by Motor Imagery Involving a Paretic Wrist and Hand for Upper-Extremity Motor Improvement in Severe Stroke: A Preliminary Study

**DOI:** 10.3390/brainsci13010069

**Published:** 2022-12-29

**Authors:** Tianhao Gao, Yiqian Hu, Jie Zhuang, Yulong Bai, Rongrong Lu

**Affiliations:** 1 Department of Rehabilitation Medicine, Huashan Hospital, Fudan University, Shanghai 200040, China; 2 School of Psychology, Shanghai University of Sport, Shanghai 200438, China; 3 National Center for Neurological Disorders, Shanghai 200040, China

**Keywords:** stroke, motor imagery, brain–computer interface, virtual reality, rTMS, DTI

## Abstract

Approximately two-thirds of stroke survivors experience chronic upper-limb paresis; however, treatment options are limited. Repetitive transcranial magnetic stimulation (rTMS) can enhance motor function recovery in stroke survivors, but its efficacy is controversial. We compared the efficacy of stimulating different targets in 10 chronic stroke patients with severe upper-limb motor impairment. Motor imagery-based brain–computer interface training augmented with virtual reality was used to induce neural activity in the brain region during an imagery task. Participants were then randomly assigned to two groups: an experimental group (received high-frequency rTMS delivered to the brain region activated earlier) and a comparison group (received low-frequency rTMS delivered to the contralesional primary motor cortex). Behavioural metrics and diffusion tensor imaging were compared pre- and post rTMS. After the intervention, participants in both groups improved somewhat. This preliminary study indicates that in chronic stroke patients with severe upper-limb motor impairment, inducing activation in specific brain regions during motor imagery tasks and selecting these regions as a target is feasible. Further studies are needed to explore the efficacy of this intervention.

## 1. Introduction

Stroke is the second most common cause of death globally, and its prevalence is projected to increase in the coming years in parallel with an increase in life expectancy [1]. Notwithstanding considerable improvements in managing the acute phase of stroke, some residual disability persists in most patients, necessitating rehabilitation [2], which incurs a heavy economic burden on families and society [3]. Hemiplegia is the most common impairment following a stroke [4,5], and approximately 37–50% of stroke survivors live with chronic severe upper-limb paresis, characterised by limited active range of motion (AROM), limited strength, impaired coordination from the shoulder to the hand and fingers, and severely diminished ability to perform activities of daily living (ADL) [6]. Therefore, rehabilitation interventions that are both effective and applicable for recovery from severe upper-limb motor impairment are an urgent clinical need.

Transcranial magnetic stimulation (TMS) is a brain stimulation technique that modulates brain activity noninvasively. This is accomplished by inducing electrical currents via rapidly changing magnetic field pulses. When TMS is applied in repetitive trains of stimulation, i.e., repetitive TMS (rTMS), its effects on cortical excitability can outlast the period of stimulation [7]. Two general types of rTMS protocols are used in stroke rehabilitation. The first is excitatory high-frequency (HF) rTMS stimulation, which is applied over the ipsilesional primary motor cortex (M1) or adjacent brain areas. The mechanism behind these protocols may strengthen synaptic connections in descending motor pathways [8]. The second protocol involves applying low-frequency (LF) rTMS over a contralesional M1, which may reduce the interhemispheric inhibition exerted by the contralesional M1 on the ipsilesional M1, thus promoting cortical reorganisation in the ipsilesional hemisphere. Both protocols have been reported to improve motor recovery in post-stroke patients [8]. However, the inter-individual variability of the responses to rTMS intervention remains high. Several studies [9,10] have investigated the efficacy of rTMS in promoting the recovery of upper-limb motor function in stroke patients, but the results are contradictory.

The question of how to precisely select the stimulation target is presently one of the most concerning issues in this space. In previous studies, the stimulated target and the protocol were heterogeneous. Some studies [11] indicate that HF-rTMS may contribute more to the functional connectivity reorganisation of the ipsilesional motor network and realise greater benefit to motor recovery than LF-rTMS. Other studies [12] have indicated that LF-rTMS has a positive effect on grip strength and lower-limb function, as assessed using the Fugl–Meyer Assessment (FMA) scale. Presently, however, applying LF-rTMS to the contralesional M1 for hand motor recovery in the post-acute stage of stroke is recommended based on level A evidence (“definitely effective or ineffective”), and HF-rTMS of the ipsilesional M1 is recommended based on level B evidence (“probably effective or ineffective”) [13]. However, in some stroke patients with severe brain injury, motor-evoked potentials (MEPs) cannot be recorded on the affected side of the brain; consequently, the stimulus target cannot be precisely determined. Furthermore, the residual function of the injured cortex may not be sufficient to dominate the paretic extremity for the completion of simple activities [14]. In contrast, the results of other studies indicate that, among these stroke patients, HF-rTMS over the contralesional side may improve motor function to some degree [9,11]. Because the accuracy of this neural modulation technique is correlated with the outcome of this intervention, the choice of stimulation target is crucial. Therefore, it is worth researching whether such a functional area exists in stroke patients with severe motor impairment and, if it exists, whether HF-rTMS over this region can further improve the motor function of a paretic wrist and hand.

In chronic stroke patients, a recent model known as the “bimodal-balance recovery” hypothesis has attempted to define the role of contralesional and ipsilesional cortices [15]. This hypothesis highlights the role of contralesional motor cortices varied based on the amount of ipsilesional reserve and neural pathways available to contribute to recovery. In patients with mild motor impairment, contralesional influence is believed to be in inhibitory, whereas in patients with severe motor impairment, the contralesional influence is thought to be supportive for paretic limb motor function. More recently, Lin et al. [16] further investigated the relationship between interhemispheric balance and motor performance and confirmed the above hypothesis. Therefore, it is worth further investigating the role of different hemispheres in chronic stroke patients. Moreover, in chronic stroke patients with severe upper-limb motor impairment, it is difficult to locate the brain regions activated by a motor task focused on the affected wrist and hand because there is no actual movement of the paretic wrist and hand. However, motor imagery ability is retained even in patients with severe motor impairment [17]. Furthermore, several studies have revealed that motor imagery possesses many of the same properties—in terms of temporal regularities, programming rules, and biomechanical constraints—observed in the corresponding real action [18,19]. In our previous research [20,21], we also found that even stroke patients with severe upper-limb motor impairment could elicit activation of the associated brain regions during motor imagery involving their paretic wrist and hand using a motor imagery-based brain–computer interface (BCI) with different end effectors. After several sessions of BCI training, the motor imagery (MI)-related electroencephalogram (EEG) activity had more discriminable patterns. These changes gradually converged, appearing predominantly in the centro-parietal cortical region (e.g., C3 and C4). Considering the aforementioned theoretical basis, we further investigated whether HF-rTMS stimulation applied over the brain regions activated during MI would improve motor function of the paretic upper limb in stroke patients. In this study, we first had stroke patients with severe upper-limb motor impairment undergo MI-based BCI training augmented with virtual reality to induce neural activity in the brain region typically activated during MI tasks. Subsequently, HF-rTMS was delivered to this activated brain region. This preliminary study investigates the feasibility and efficacy of this stimulation protocol.

## 2. Materials and Methods

### 2.1. Participants

This study is a randomised, parallel, controlled, single-blinded clinical trial. Ten stroke patients who had suffered a stroke at least six months previously and continued to experience severe chronic upper-limb motor impairment were recruited for this preliminary study. A clinical assessment of the motor impairment of the participants was performed by a physiatrist who was unaware of the randomisation assignment of the participants. The inclusion criteria were as follows: (1) aged 18–90 years old at the time of randomisation; (2) more than 6 months since their first clinical cortical or subcortical, ischaemic, or haemorrhagic stroke, confirmed via computed tomography (CT) or magnetic resonance imaging (MRI); (3) no active extension of the paretic wrist and scores of grade 0–1 on the manual muscle test (MMT) for wrist extension; (4) no cognition impairment, with a Montreal Cognitive Assessment (MOCA) score of ≥26; and (5) no hearing or visual impairments. The exclusion criteria included the following: (1) patients with contraindications for MRI or rTMS, (2) participation in other clinical trials, and (3) pregnancy. The study was approved by the Ethics Committee of Huashan Hospital and was conducted in accordance with the tenets of the Declaration of Helsinki. All participants provided written informed consent before participating. This study was registered with the China Clinical Trial Registration Centre (registration number: ChiCTR2000036423).

### 2.2. Intervention

**Figure 1:**
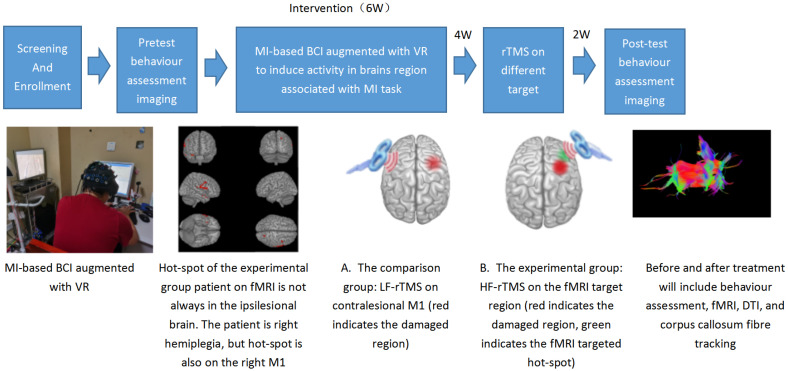
Overview of study design showing the timeline, intervention, and outcome measures. MI, motor imagery; BCI, brain–computer interface; VR, virtual reality; rTMS, repetitive transcranial magnetic stimulation; LF, low frequency; HF, high frequency; fMRI, functional magnetic resonance imaging; DTI, diffusion tensor imaging.

The intervention was divided into two phases. The first phase involved inducing the brain regions typically activated during MI involving the paretic wrist and hand using an MI-based BCI system augmented with virtual reality (VR). In the second phase, rTMS was used to stimulate the brain, with the participants randomly divided into two groups: an experimental group and a comparison group. The stimulation target of the experimental group was the aforementioned brain regions activated by MI-based BCI augmented with VR, while the comparison group received an LF-rTMS intervention applied over the contralateral M1. An overview of the study design showing the timeline, intervention, and measured outcomes is presented in Figure 1.

#### 2.2.1. Inducing Neural Activity in Associated Brain Regions during Motor Imagery Involving a Paretic Wrist and Hand 

An 11-channel, high-resolution EEG system, g.USBamp (g.tec Medical Engineering, Schiedlberg, Austria), was used for this study. The electrodes were attached to the scalp, per the 10–20 international electrode placement system, as follows: FC3, FC4, C5, C3, C1, CZ, C2, C4, C6, CP3, and CP4. The ground electrodes were placed on the medial frontal cortex. The reference electrodes were fixed at the left and right mastoids, and the average value from the bilateral electrodes was used as the reference. The EEG signals were collected at a sampling rate of 256 Hz. 

This process is described in detail in our previous research [20]. Each session lasted 30 min (four cycles of six minutes each, with two-minute intervals) and was conducted five days a week for four weeks.

#### 2.2.2. Activated Brain Regions during MI Tasks Determined Using fMRI

**Figure 2:**
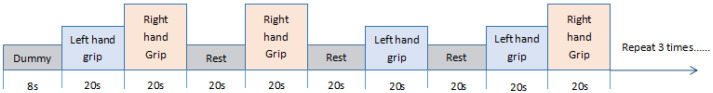
fMRI tasks.

fMRI was performed to determine the brain regions activated during the MI task. We used a block design with three tasks: A is a prompt to imagine the grasping movement of the left hand, B is a prompt to imagine the grasping movement of the right hand, and C is a prompt to rest. Each task lasted for 20 s. During the MI task, short videos of the grasping movement of the left hand, the grasping movement of the right hand, and a blank screen were shown to the participants. The three tasks were performed in the order ABC, BCA, and CAB, and each sequence was repeated three times (Figure 2). The participants were instructed to do the following: (1) mentally imagine the action of grasping using their left/right hand following the video instruction and (2) just rest without any action or imagery when presented with blocks of blank screen.

The participants were scanned in a 3.0 Tesla Siemens MAGNETOM Prisma whole-body 60 cm bore human scanner equipped with 80 mT/m gradients and a 200 T/m/s slew rate (Siemens Healthineers, Erlangen, Germany) at Shanghai University of Sport. We used an eight-channel head coil for radio frequency (RF) transmission and reception. We collected sagittal T1-weighted images as the localiser and performed a semiautomated high-order shimming programme to ensure global field homogeneity. A three-dimensional fast spoiled gradient echo pulse sequence was chosen for acquiring high-resolution structural images with the following parameters: repetition time (TR) = 8.156 s, inversion time (TI) = 450 ms, echo time (TE) = 3.18 ms, voxel size = 1 × 1 × 1 mm^3^, 166 contiguous slices, field of view (FOV) = 25.6 cm^2^, flip angle = 12°, sense factor = 2. A single session of functional images, which are sensitive to blood oxygen level-dependent (BOLD) contrast, were acquired using an echo planar imaging (EPI) sequence (TR = 2 s, TE = 30 ms, voxel size = 3 × 3 × 3 mm^3^, FOV = 19.2 cm^2^, flip angle = 90°, SENSE factor = 1, 42 contiguous oblique axial slices parallel to the anterior commissure–posterior commissure line, interleaved acquisition) before and after BCI training. Three initial RF excitations were performed to achieve steady state equilibrium, and these were excluded from subsequent analyses. 

Preprocessing was carried out using statistical parametric mapping (SPM) version 12 (SPM12; Wellcome Institute of Cognitive Neurology, London, UK) running under MATLAB (Mathworks Inc., Natick, MA, USA). In each functional session, all EPI images were realigned to the first EPI image to correct for head motion, followed by slice time correction, co-registration between functional images and structural images, and spatial normalisation to a standard Montreal Neurological Institute (MNI) EPI template. A cut-off of 25 mm was chosen for discrete cosine transform functions, and all normalised EPI images were smoothed with a 6 mm full-width half-maximal Gaussian smoothing kernel. Statistical modelling was performed using a general linear model implemented in SPM12. To minimise potential nuisance variables in comparisons across sessions, the two functional sessions were concatenated into a single session per the procedures in previous research.

For the fixed-effect analysis, the design matrix comprised the following independent events: MI of left hand, MI of right hand, rest (null event) before intervention, MI of left hand, MI of right hand, and rest after intervention together with a set of linear trend predictors, six head motion parameters, and a confound-mean predictor. To detect neural activation of the entire block, each 20 s block was modelled using a canonical hemodynamic response function (HRF), and the onset of each block was taken as the onset of the block in the SPM analysis model with a duration of 20 s.

Significant activations were thresholded at *p* < 0.001, voxel-level uncorrected and at *p* < 0.05, cluster-level corrected, and for multiple comparisons unless otherwise stated. The SPM coordinates are reported in the MNI space. Brain regions were identified using the automated anatomical labelling (AAL) atlas [22] and Brodmann templates, as implemented in MRIcron.

#### 2.2.3. Transcranial Magnetic Stimulation

##### Measurement of Cortical Excitability

We used MEG-TD (Wuhan Yiruide Medical Equipment New Technology Co., Ltd., Wuhan, China) in this study. MEG-TD generates a bidirectional pulse waveform with a pulse width of 340 ± 20 μs and the pulse rise time of 60 ± 10 μs. 

Electromyography (EMG) data were recorded from the first dorsal interosseous (FDI) using standard Ag/AgCl electrodes and a ground electrode positioned on the wrist. The EMG signals were amplified with a band pass filter of 10 Hz to 2 kHz. Because the participants in our study had no detectable MEP in the lesioned hemisphere, the motor threshold and MEP of the contralesional hemisphere were recorded. To determine the resting motor threshold (RMT), TMS was administered a commercially available figure-of-eight coil (YRD, maximum magnetic field intensity = 2 T, diameter = 9 cm; Wuhan Yiruide Medical Equipment New Technology Co., Ltd., Wuhan, China) using MEG-TD. The coil was placed in a tangent direction to the head, with the centre towards stimulating target. The RMT was assessed per the guidelines of the International Federation for Clinical Neurophysiology, and the minimum TMS intensity capable of producing at least five MEPs of 50 μV amplitude in 10 consecutive stimuli was estimated [23]. TMS intensity was adjusted to achieve an MEP of 1 mV peak-to-peak amplitude in the FDI muscle, and 10 consecutive MEPs were subsequently recorded.

##### Repetitive TMS

The 10 participants were randomly divided into two groups, with five participants in each group. rTMS was delivered according to the group assignment using MEG-TD with a figure-of-eight coil. For the comparison group, LF-rTMS was delivered to the contralesional M1 per the stimulation protocol recommended by the guideline (100 pulses of 1 Hz stimulation per session, with a 1 s interval between sessions and 12 sessions per treatment, totaling 1200 pulses at 80% rMT) [13]. In the experimental group, HF-rTMS was delivered to the brain regions activated during the MI task. We first determined the associated brain regions using fMRI and then converted the fMRI data, per the 10–20 international electrode placement system, to locate the stimulation target. The stimulation scheme was 10 Hz stimulation for 3 s per session, with an 8 s interval between sessions, 30 sessions per treatment, totalling 1200 pulses at 100% rMT. rTMS was conducted once a day, five times a week for 10 times in total.

### 2.3. Assessments

#### 2.3.1. Primary Outcome

The change in upper-limb motor impairment at the end of the treatment was assessed using the motor status scale (MSS). MSS measures shoulder, elbow (maximum score = 40), wrist, hand, and finger movements (maximum score = 42), and it affords a reliable and valid assessment of upper limb impairment and disability following a stroke [24]. 

#### 2.3.2. Secondary Outcomes

Secondary measures included the FMA scale (used for the upper extremities, range of motion, or wrist motor function) and the action research arm test (ARAT).

#### 2.3.3. Commissural Fibres across the Corpus Callosum

Diffusion tensor imaging (DTI) was also performed to assess the white matter integrity of the fibres across the corpus callosum. Fractional anisotropy (FA) was measured at the corpus callosum because it is one of the most important white matter structures in the brain; the corpus callosum connects the two cerebral hemispheres and transmits information between them [25]. Previous research indicates that the anisotropy of the corpus callosum may be corelated with motor impairment and with functional gains following rehabilitation intervention [26,27].

DTI analysis was performed using the software library of the Oxford Centre for Functional Magnetic Resonance Imaging of the Brain. Skull-stripped DTI images were registered to b = 0 images to correct for eddy current distortions and simple head motion. Diffusion tensors were fitted to each voxel of the diffusion-weighted images, and Markov chain Monte Carlo sampling was used to build up distributions at these voxels. The resulting DTI images were then co-registered to the T1-weighted anatomical images. For fibre tracking, we adapted the two-step fibre-tracking method described by Wahl [28]. First, we placed a rectangular region of interest (ROI) in the primary motor regions of the precentral gyri (M1) of both hemispheres. Following the tracking step, a second ROI on the corpus callosum was added where the fibres from the first tracking emerged, and a second tracking was performed. After obtaining the DTI data, the FA value of the entire transcallosal motor tract was determined. The assessments were conducted before and after rTMS intervention.

### 2.4. Statistical Analysis

The Statistical Package for the Social Sciences (SPSS) version 20.0 (IBM, Chicago, IL, USA) was used to complete the statistical analysis. Considering the small sample size of this study, Wilcoxon signed-ranks test was used for within-group comparison before and after the intervention. Mann–Whitney U-test was used for between-group comparison. The level of significance was set at *p* < 0.05.

## 3. Results

### 3.1. Participants

We recruited ten participants: nine male and one female, and all ten participants were right-handed. Demographic information is presented in Table 1. All participants completed the two phases of the intervention and all the assessments. All the participants were assessed with the kinaesthetic and visual imagery questionnaire (KVIQ) [29], and all of their scores were above 25, which indicated they could actually perform motor imagery.

### 3.2. Activated Brain Regions during Motor Imagery Tasks and the Stimulation Target

fMRI was performed before and after MI-based BCI augmented with VR to determine the activated regions during MI tasks involving the paretic wrist and hand. The characters of each participant and the stimulating target of the experimental group are presented in Table 2. 

### 3.3. Behavioural Outcome Metrics

There was no significant difference between the two groups for all measured behavioural outcomes before the intervention (Table 3). Compared with the pre-rTMS scores, the MSS scores of both groups (experimental group: pre 14.72 ± 6.01; post 16.72 ± 7.14; comparison group: pre 14.04 ± 6.07; post 14.88 ± 6.42) improved, but there was no significant improvement (*p* = 0.066 and *p* = 0.109). There was also no significant difference when comparing the two groups. 

**Figure 3:**
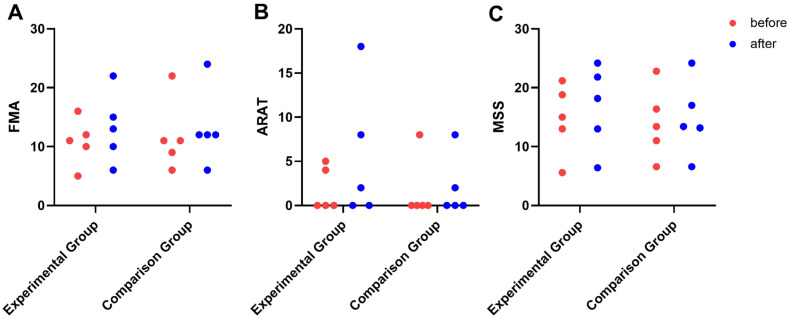
Behavioural Outcomes in Experimental group and Comparison group. (**A**) For FMA; (**B**) for ARAT; (**C**) for MSS.

After the intervention, the FMA scores of both groups improved to a certain extent; however, there was no significant difference within and between the groups. All the participants could not flex or extend their wrists at baseline, but they regained some AROM after the rTMS intervention. For the experimental group, the AROM for wrist flexion was 16.00 ± 26.08 after the rTMS intervention, while it was 6.00 ± 8.94 for the comparison group. There was also no change in the ARAT scores, which indicate the practical functional capacity of the hand. The changes in the measured behavioural outcomes are presented in Figure 3.

### 3.4. Commissural Fibres across the Corpus Callosum

FA was measured at the corpus callosum before and after the rTMS intervention, and there was no significant difference between the FA values of the two groups. After the rTMS intervention, FA in the experimental group showed a trend of increase, while the FA values of the comparison group did not change significantly (Table 4).

## 4. Discussion

Rehabilitation is critical for reducing stroke-related disability [30], and there is growing recognition that cortical neuroplasticity supporting adaptive recovery may extend for years after stroke [31]. However, up to 50% of stroke survivors still have persistent, severe upper-extremity paresis even after receiving rehabilitation treatment. TMS is a safe, non-invasive method of stimulating the cerebral cortex [32]. When used at low or high frequencies, rTMS may potentially enhance the ability of the brain to relearn task-specific functions as well as augment the effects of rehabilitation via modulating corticomotor excitability [33]. However, the reported efficacy of this intervention differs significantly. Furthermore, in light of the negative results from the NICHE trial [34], evidence for the efficacy of LF-rTMS to the contralesional M1 for motor recovery during the chronic stage of stroke is controversial. Meanwhile, the conventional approach of facilitating excitability of the ipsilesional primary motor cortex also fails to produce motor improvement in stroke survivors with severe loss of ipsilesional substrate [35]. Previous studies indicate that different stimulation targets may affect the efficacy of rTMS for the recovery of upper-limb motor function differently [36]. Therefore, it is worth considering how to select appropriate targets before rTMS intervention is applied. In this study, the brain regions activated by MI were selected as the intervention target, and we investigated the effect of stimulating this target on the recovery of upper-limb motor function in chronic stroke patients with severe upper-limb motor impairment.

Our results indicate that MI ability is retained even in stroke patients with severe motor impairment, and this ability can be further enhanced after specific feedback training, which is consistent with the findings of previous research [17], including our previous study [20]. Therefore, in stroke patients with severe motor impairment, selecting the brain regions activated during MI tasks as rTMS intervention targets is feasible and has certain potential value. Our preliminary results also confirm the feasibility of identifying intervention targets using this approach. In addition, we further compared this new target with the conventional target [13]. After the intervention, the MSS scores of both groups increased, but there was no significant difference between and within the two experimental groups. The results of other behavioural assessments also indicate some improvement, but the improvements were not statistically significant. For this study, we recruited stroke patients in the chronic stage. Brain plasticity in this stage might experience a more complicated reconstruction and follow other recovery patterns [37]. In a chronic stroke brain, there may be a new functional cerebral architecture, one that is not as effective as that in the intact brain but still attempts to generate some form of motor signal to the downstream neurons in the most effective way it can. Both the ipsilesional and contralesional motor sensory regions may be involved in this process [38]. Lin et al. [16] confirmed that balance and recovery have a bimodal dependence. They also identified a threshold of the clinical score useful to stratify stroke patients (UEFM = 43). Above this threshold, better motor performance is associated with low transcallosal inhibition from the contralesional hemisphere, while below this threshold, better performance is associated with higher transcallosal inhibition. In our study, we recruited patients whose UEFM were all below 43. As Lin et al. indicated in their study, the contralesional hemisphere might play a supportive role in the recovery of the aforementioned participants. However, in our results, we did have participants (2,3,4) that showed the recovery pattern that Lin et al. pointed out in their study. Still, participants (1,5) showed the activation of ipsilesional brain regions during motor imagery task, which was contrary to the results of Lin et al.’s study. This indicates that there are different types of brain remodelling in chronic stroke patients. Therefore, an individualised target selection may further increase the therapeutic effect of rTMs. Considering the preliminary nature and small sample size of our study, we could not make a concrete conclusion. Further studies should be performed to investigate the relationship between the two hemispheres in chronic stroke patients.

In this study, we converted the activated brain regions determined using fMRI per the 10–20 international electrode placement system. Although it would be more precise to locate the target using an rTMS navigator, considering the coverage of the navigator, it is more feasible to perform the conversion per the 10–20 international electrode placement system for rTMS stimulation.

We also traced the transcallosal fibres in our participants. In healthy individuals, interhemispheric neural activity between the homologous motor cortices is well-balanced through the opposing inhibitory influences exerted by the M1s of both hemispheres [39]. Previous research indicates that increased transcallosal fibre microstructure may be predictive of the interhemispheric inhibitory capacity in healthy individuals [40]. Investigating fibres via the corpus callosum can reflect brain plasticity from a structural perspective. In this study, we found that FA increased in the experimental group (*p* = 0.059) after rTMS intervention. Because our participants were stroke patients with severe upper-limb impairment, the FA of fibres across the corpus callosum may primarily reflect fibres from the contralesional M1 to the ipsilesional M1. With improvements in upper-limb motor function, the increased FA may indicate the specific role of the contralesional M1 in recovery from severe brain injury. These results are also in agreement with the findings of the study by Grefkes [41], in which they concluded that movement of a stroke-affected hand showed additional inhibitory influences from the contralesional to ipsilesional M1 that correlated with the degree of motor impairment. Further studies are needed to confirm the effect of rTMS delivered to different brain regions and how it affects the balance between the two hemispheres and the recovery from motor impairment.

There are some limitations to this study. First, this is a preliminary study, and only 10 participants were recruited, which certainly impacts its efficacy. Second, in this study, there were exactly 10 sessions of rTMS interventions. Whether increasing the number of intervention sessions will further increase the efficacy of rTMS treatment also needs to be verified in future studies. Third, although all participants underwent task-oriented training focused on the upper limbs, we did not further define the implementation time of the training. In future research, we will implement task-oriented training immediately after rTMS, which may yield the benefit of improving recovery. Therefore, we will increase the sample size and optimise the intervention plan based on the results of this study and continue to conduct randomised, controlled studies to investigate the effect of using task-activated brain regions as the intervention target for the improvement of upper-limb motor function in chronic stroke patients with severe upper-limb motor impairment.

## 5. Conclusions

In chronic stroke patients with severe upper-limb motor impairment, determining the brain regions activated during an MI task and selecting them as the target of an rTMS intervention is feasible. Due to the preliminary nature of this study, further studies are needed to explore the efficacy of this intervention.

## Figures and Tables

**Table 1:** Demographic characteristics of the participants (n = 10).

Participant	Group	Sex	Age (y)	Diagnosis	Affected UE	Post-Stroke Duration (mo)
1	Experimental	M	43	Haemorrhagic	Left	8
2	Experimental	M	68	Ischaemic	Left	16
3	Experimental	M	42	Haemorrhagic	Left	20
4	Experimental	M	75	Ischaemic	Left	6
5	Experimental	F	58	Haemorrhagic	Right	7
6	Comparison	M	65	Haemorrhagic	Left	11
7	Comparison	M	32	Haemorrhagic	Left	6
8	Comparison	M	56	Ischaemic	Right	8
9	Comparison	M	66	Ischaemic	Left	7
10	Comparison	M	41	Haemorrhagic	Left	20

M, male; UE, upper extremity; y. year; mo, month.

**Table 2:** Characteristics of each participant and the stimulating target of the experimental group.

	Affected Hemisphere	Activated Brain Regions in fMRI	Activated Brain Regions Converted According to 10–20 International System	Activated Brain Regions
Participant 1	Right	Mainly in the right premotor cortex (BA 6) and precentral gyrus (M1, BA 4)	FC4	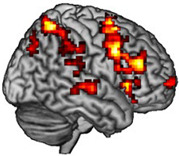
Participant 2	Left	Mainly in the right precentral gyrus (M1, BA 4), premotor cortex, and supplementary motor cortex (SMA, BA 6)	C2	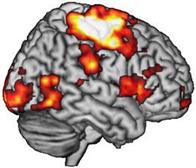
Participant 3	Right	Mainly in the left precentral gyrus (M1, BA 4) and premotor cortex (BA 6)	C3	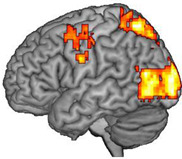
Participant 4	Right	Mainly in the left precentral gyrus (M1, BA 4) and premotor cortex (BA 6)	C1	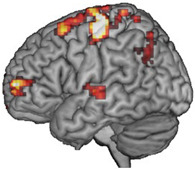
Participant 5	Right	Mainly in the right precentral gyrus (M1, BA 4) and premotor cortex (BA 6)	C1	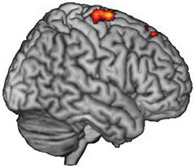

**Table 3:** Behavioural Outcome Metrics.

Participant	Group	Pre MSS	Post MSS	Pre FMA	Post FMA	Pre ARAT	Post ARAT	Pre AROM Flexion	Post AROM Flexion	Pre AROM Extension	Post AROM Extension
1	Experimental	18.80	21.80	12.00	15.00	4.00	18.00	0°	0°	0°	0°
2	Experimental	13.00	13.00	10.00	10.00	0.00	0.00	0°	0°	0°	0°
3	Experimental	15.00	18.20	11.00	13.00	0.00	2.00	0°	20°	0°	0°
4	Experimental	21.20	24.20	16.00	22.00	5.00	8.00	0°	60°	0°	25°
5	Experimental	5.60	6.40	5.00	6.00	0.00	0.00	0°	0°	0°	0°
6	Comparison	16.40	17.00	11.00	12.00	0.00	0.00	0°	20°	0°	0°
7	Comparison	22.80	24.20	22.00	24.00	8.00	8.00	0°	10°	0°	20°
8	Comparison	11.00	13.20	9.00	12.00	0.00	2.00	0°	0°	0°	0°
9	Comparison	6.60	6.60	6.00	6.00	0.00	0.00	0°	0°	0°	0°
10	Comparison	13.40	13.40	11.00	12.00	0.00	0.00	0°	0°	0°	0°

**Table 4:** FA for fibres across corpus callosum.

	Experimental Group		Comparison Group				
	Baseline	Post rTMS	Baseline	Post rTMS	Sig. ^a^	Sig. ^b^	Sig. ^c^
FA	0.53 ± 0.05	0.56 ± 0.06	0.55 ± 0.04	0.56 ± 0.06	0.059	0.828	0.909

^a,b^ FA (pre–post), within-group comparisons in experimental group and comparison group, respectively. ^c^ FA between groups.

## Data Availability

The data presented in this study are available on request from the corresponding author.
